# DCL-A: An Unsupervised Ultrasound Beamforming Framework with Adaptive Deep Coherence Loss for Single Plane Wave Imaging

**DOI:** 10.3390/diagnostics15243193

**Published:** 2025-12-14

**Authors:** Taejin Kim, Seongbin Hwang, Minho Song, Jinbum Kang

**Affiliations:** 1 Department of Computer Science and Information Engineering, The Catholic University of Korea, Bucheon 14662, Republic of Korea; tjkime1903@catholic.ac.kr; 2 Department of Artificial Intelligence, The Catholic University of Korea, Bucheon 14662, Republic of Korea; sean1675@catholic.ac.kr; 3 Department of Radiology, Standford University, Standford, CA 94305, USA; minhos@stanford.edu; 4 Department of Biomedical Software Engineering, The Catholic University of Korea, Bucheon 14662, Republic of Korea; 5 Department of Healthcare and Artificial Intelligence, The Catholic University of Korea, Bucheon 14662, Republic of Korea

**Keywords:** ultrasound imaging, deep neural network, unsupervised learning, adaptive deep coherence loss

## Abstract

**Background/Objectives**: Single plane wave imaging (SPWI) offers ultrafast acquisition rates suitable for real-time ultrasound imaging applications; however, its image quality is compromised by beamforming artifacts such as sidelobe and grating lobe interferences. **Methods**: In this paper, we introduce an unsupervised beamforming framework based on adaptive deep coherence loss (DCL-A), which employs linear ($\alpha_{\mathrm{linear}}$) or nonlinear weighting ($\alpha_{\mathrm{nonlinear}}$) within the coherence loss function to enhance the artifact suppression and improve overall image quality. During training, the adaptive weight (*α*) is determined by the angular distance between the input and target PW frames, assigning lower α values for smaller distances and higher α values for larger distances. Therefore, this adaptability enables the method to surpass conventional DCL (no weighting) by emphasizing the different spatial correlation characteristics of mainlobe and sidelobe signals. To assess the performance of the proposed method, we trained and validated the network using publicly available datasets, including simulation, phantom and in vivo images. **Results**: In the simulation and phantom studies, the DCL-A with $\alpha_{\mathrm{nonlinear}}$ outperformed the comparison methods (i.e., conventional DCL and DCL-A with $\alpha_{\mathrm{linear}}$) in terms of peak range sidelobe level (PRSLL), achieving 7 dB and 14 dB greater sidelobe suppression, respectively, while maintaining a comparable full width at half maximum (FWHM). In the in vivo study, it achieved the highest contrast resolution among the comparison methods, yielding 2% and 3% improvements in generalized contrast-to-noise ratio (gCNR), respectively. **Conclusions**: These results demonstrate that the proposed deep learning-based beamforming framework can significantly enhance SPWI image quality without compromising frame rate, indicating promising potential for high-speed, high-resolution clinical applications such as cardiac assessment and real-time interventional guidance.

## 1. Introduction

Ultrasound imaging has become one of the most widely used clinical imaging modalities owing to its real-time capability, noninvasive nature, and relatively low cost. Conventional ultrasound systems employ focused transmission, sequentially emitting focused beams along each scanline to achieve high image quality. Recently, plane-wave imaging (PWI), which allows ultrafast frame rates (≥1 kHz) by transmitting PWs, has been introduced; however, it still suffers from the restriction that multiple PW transmissions at different angles are required to enhance image quality [1,2,3]. To address this issue, single plane wave imaging (SPWI), which reconstructs the entire image from a single PW transmission, has been proposed more recently. However, its image quality remains relatively low due to the low signal-to-noise ratio (SNR) and beamforming artifacts such as sidelobe and grating lobe interferences [1,2,3,4,5].

To overcome these limitations in SPWI, various rule-based adaptive beamforming techniques based on signal coherence characteristics or statistical properties of radiofrequency (RF) signals have been proposed [6,7,8,9]. Recently, the delay-multiply-and-sum (DMAS) beamforming method employing inter-channel signal correlation exhibited great performance in reducing sidelobe and grating lobe artifacts [10]. However, it is susceptible to signal distortion due to the nonlinear nature of the multiplication operation, which can introduce dark region artifacts (DRAs) and consequently degrade contrast resolution [11,12]. More recently, with the advances in artificial intelligence (AI) technology, deep learning (DL)-based approaches have been introduced to improve image quality in PWI or SPWI [13,14,15,16,17,18,19,20,21,22]. Early studies have primarily focused on post-processing methods based on convolutional neural networks (CNNs), which utilizes image-to-image translation to enhance image quality [13,14,15]. Subsequently, more fundamental end-to-end deep learning beamformers that directly process RF channel data as input have been proposed combining with GoogLeNet and U-Net architecture [16,17,18,19]. In parallel, generative adversarial networks (GANs) have been applied to convert low-quality PWI images into higher-quality images comparable to conventional focused imaging [20]. Furthermore, a cascaded DL framework combining a fully CNN with a conditional GAN has been developed to enhance SPWI performance [21], where high-quality target images were generated from PWI with a large of PW transmissions (e.g., *N* = 75). Although these supervised learning-based approaches have shown substantial performance improvements over conventional rule-based techniques, they still face inherent limitations, as their performance cannot exceed the quality of the ground-truth data. Moreover, obtaining reliable ground-truth data is particularly challenging in ultrasound imaging, as the underlying transfer function is inherently underdetermined.

To overcome these limitations of supervised learning-based approaches, fully unsupervised learning-based beamforming technique based on deep coherence loss (DCL) has recently been proposed [22]. In the DCL method, a deep neural network was trained by a unique loss function, which maximizes signal coherence (e.g., main-lobe signals) between multiple PW data without requiring ground truth images; the trained DL model encourages high-quality PWI from low-quality SPWI data. In addition, this universal beamformer based on complex baseband signals supports real-time inference while maintaining image quality comparable to 75 PW imaging using only a single transmission. However, the DCL may be limited in distinguishing the subtle correlations of signal characteristics that occur across different steering angles in a set of PW data since the coherence loss function assigns uniform weights to all PW angles during training. For instance, highly correlated sidelobes adjacent to the main-lobe in point spread functions (PSFs) corresponding to small-angle pairs in the PW set are often difficult to fully suppress, whereas grating lobes or sidelobes associated with large-angle pairs can be more easily reduced due to their lower spatial correlation. Therefore, assigning identical weights to all PW angles may limit the performance of artifact suppression and significantly increase the training time required to achieve satisfactory performance.

Unlike the conventional DCL, which does not account for different spatial correlations among different PW angles (frames) during training, this paper presents an unsupervised beamforming framework based on an adaptive deep coherence loss (DCL-A). In DCL-A, the adaptive weight (α) is determined by the angular distance (d) between the input and target PW frames, assigning lower α values for smaller angular distances and higher α values for larger angular distances. As a result, the highly correlated sidelobe components between adjacent frames (small d) are more effectively suppressed by lower weights, whereas the more distinct mainlobe components between distant frames (large d) are emphasized through higher weights. To further evaluate the degree (gradient) of weighting with respect to the angular distance, linear ($\alpha_{\mathrm{linear}}$) or nonlinear ($\alpha_{\mathrm{nonlinear}}$) weighting factors are applied to the unique coherence loss function according to the PW angles in a set of PW data. This adaptive weighting strategy is fundamentally different from prior loss-function modifications (e.g., weighted cross-entropy [23] and focal loss [24]), which are inherently designed for supervised learning frameworks. Therefore, compared to conventional DCL-based methods, the proposed approach achieves further improvements in main-lobe enhancement and sidelobe suppression, thereby enhancing overall SPWI image quality without sacrificing the high frame rate. To assess the effectiveness of the proposed method, we trained and validated the network on publicly available datasets consisting of simulation, phantom, and in vivo images.

## 2. Materials and Methods

### 2.1. Unsupervised Beamforming Framework

**Figure 1:**
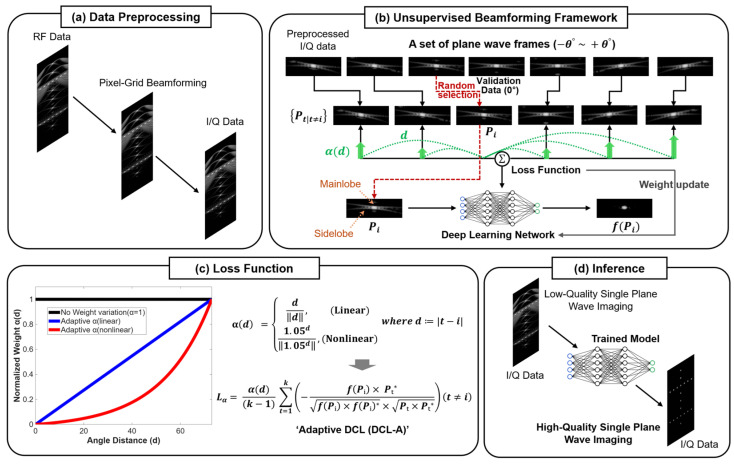
Overview of the proposed unsupervised beamforming framework with adaptive deep coherence loss (DCL-A). (**a**) Preprocessing to generate training input (I/Q data) from RF data. (**b**) Overall unsupervised beamforming framework using PWI dataset. (**c**) DCL-A loss formulation incorporating either linear ($\alpha_{\mathrm{linear}}$) or nonlinear ($\alpha_{\mathrm{nonlinear}}$) weighting as a function of frame (angular) distance ($d=\left|t-i\right|$). (**d**) Inference step using the trained model to produce high-quality SPWI images.

The proposed unsupervised deep learning–based beamforming method reconstructs high-quality SPWI images by training directly on PWI data (i.e., a set of plane-wave frames acquired from 75 PW transmissions) without requiring ground-truth images. Figure 1 represents the overview of the unsupervised beamforming framework based on adaptive deep coherence loss (DCL-A). For training data generalization, a preprocessing is performed to generate 2-channel in-phase and quadrature (I/Q) data using a pixel-grid delay calculator from radiofrequency (RF) data, enabling the model to be trained independently of specific system configurations, as illustrated in Figure 1a. Figure 1b illustrates the unsupervised beamforming framework, in which the key training objective is to learn spatial signal coherence across multiple PW frames acquired at different steering angles. In this process, signals with high inter-frame correlation corresponding to tissue structures (main lobe signals) are emphasized, while low-correlation components such as noise and artifacts (sidelobe and grating lobe signals) are suppressed. Based on this principle, the loss function (DCL-A) is computed using the remaining target frames $P_{t}$, excluding both the 0-degree validation frame and a randomly selected input frame $P_{i}$ from the set of PW frames. The resulting loss is then used to iteratively update the model parameters throughout training, as illustrated in Figure 1b. Figure 1c shows the DCL-A loss function that calculates the normalized cross-correlation between the network prediction ($f(P_{i})$) and the target data ($P_{t}$) with the adaptive weight using linear or nonlinear function. As shown in Figure 1c, the conventional DCL (black line) [22] is calculated without any weight modification across frame distances. In contrast, the proposed DCL-A integrates an adaptive weighting function into the loss, where the weight varies with the integer frame (angle) distance d. Depending on the scheme used, the weighting profile can be linear (blue line) or nonlinear (red line). After training with the DCL-A, the trained DNN model produces a high-quality SPWI image, as shown in Figure 1d.

### 2.2. Adaptive Deep Coherence Loss

Since the conventional deep coherence loss (DCL) [22] assigns identical weights to every pair of the PW dataset, it may be limited in unsupervised beamforming performance due to spatially varying correlations between PW frames. To address this limitation, we propose an adaptive deep coherence loss (DCL-A) that incorporates angle-dependent weighting. As illustrated in Figure 1b, the adaptive weight (*α*) is determined by the angular distance (d) between the input ($P_{i}$) and target ($P_{t}$) PW frames, assigning lower α values for smaller angular distances and higher α values for larger angular distances. Consequently, the highly correlated sidelobe components between adjacent frames (small d) are more effectively suppressed by lower weights, whereas the more distinct mainlobe components between distant frames (large d) are emphasized through higher weights. To further control the degree (gradient) of weighting with respect to the angular distance, a constantly increasing (linear) function and a variably increasing (nonlinear) function are compared as illustrated in Figure 1c. Therefore, in the proposed DCL-A, either a linear ($\alpha_{\mathrm{linear}}$) or nonlinear ($\alpha_{\mathrm{nonlinear}}$) weighting function is incorporated into the DCL and the adaptive weighting α can be expressed as follows:

(1) $$\alpha \left(d\right)=\left\{\begin{array}{cc} \frac{d}{\left\|d\right\|}, & \left(\mathrm{Linear}\right)\\ \frac{1.05^{d}}{\left\|1.05^{d}\right\|}, & \left(\mathrm{Nonlinear}\right) \end{array}\right.\;\;\;\mathrm{where}\;\;\;d≔\left|t-i\right|$$

where d denotes the absolute distance (scalar) between the input and the target frames ($P_{i}$ and $P_{t}$). As described in Equation (1), the linear weighting function $\alpha_{\mathrm{linear}}$ increases proportionally with the frame distance d, encouraging the network to assign higher importance to frames with larger angular spacing than to closely adjacent frames. This suggests that lower spatial coherence in large-angle PW pairs facilitates sidelobe and grating-lobe suppression, whereas the mainlobe is retained through the coherent contributions from closely spaced (smaller-angle) pairs. Moreover, the adaptive weighting effect can be further strengthened by employing a nonlinear weighting function ($\alpha_{\mathrm{nonlinear}}$), in which the weighting gradient is dramatically increased (e.g., via an exponential function) as also illustrated in Figure 1c, thereby amplifying the contrast between high- and low-coherence signal components. It should be noted that higher bases in exponential functions, which produce more dramatic weighting gradients (e.g., $y=2^{x}$), can create an imbalance in training between small angle and large angle pairs and may interfere with learning convergence due to numerical instability [25,26]. Conversely, gentler weighting gradients can approximate a linear function, in which case the performance difference may be insignificant.

Therefore, the weighting function α(d) is incorporated into the DCL term, which is formulated using the normalized cross-correlation between the input ($P_{i}$) and target ($P_{t}$) I/Q frames:

(2) $$L_{\alpha }=\frac{\alpha (d)}{\left(k-1\right)}\sum_{t=1}^{k}\left(-\frac{f\left(P_{i}\right)\times P_{t}*}{\sqrt{f\left(P_{i}\right)\times f\left(P_{i}\right)*}\times \sqrt{P_{t}\times P_{t}*}}\right)\left(t\neq i\right)$$

where k denotes the total number of PW frames acquired at different steering angles, and f represents the network prediction. The * indicates the complex conjugate of the I/Q signals. The negative sign ensures that higher coherence between the predicted and target signals leads to a lower loss value, thereby encouraging coherent beamforming during training.

### 2.3. Network Architecture

**Figure 2:**
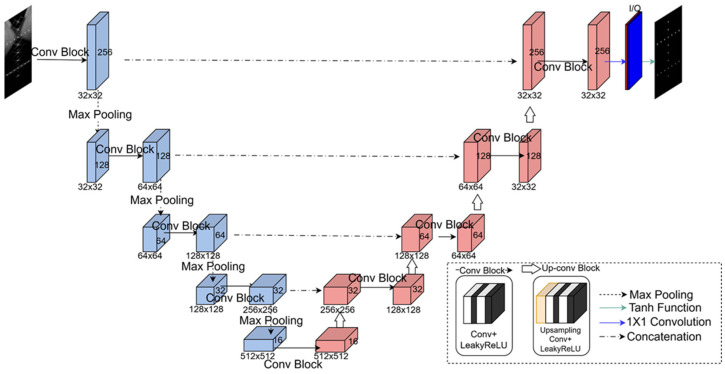
Proposed CNN model based on a U-Net architecture. The network adopts an encoder–decoder design with skip connections and employs 2-channel I/Q inputs of size 256 × 256. It comprises five encoder blocks (expanding from 32 to 512 channels) and four decoder blocks, where spatial resolution is restored via bilinear upsampling.

**Figure 3:**
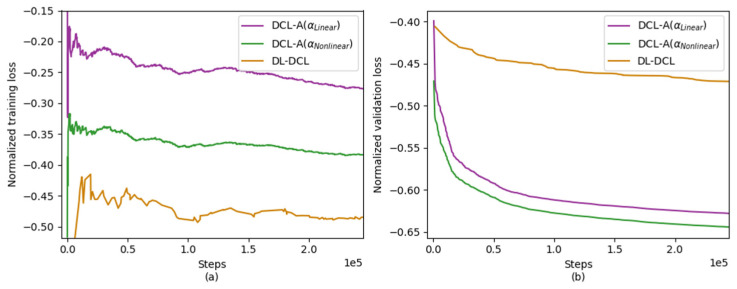
Normalized (**a**) training loss and (**b**) validation loss curves as a function of training steps (each step corresponds to a single gradient update) for the conventional DL-DCL without weighting (orange), the proposed DCL-A with $\alpha_{\mathrm{linear}}$ (purple), and the proposed DCL-A with $\alpha_{\mathrm{nonlinear}}$ (green).

The CNN model employed for training the unsupervised beamforming framework was constructed using a U-Net architecture [27]. As illustrated in Figure 2, following the preprocessing step shown in Figure 1a, the network receives a 2-channel 256 × 256 I/Q image and is trained to produce an enhanced I/Q output with the same spatial size. The encoder path (blue blocks) is formed by five convolutional blocks that progressively increase the feature depth from 32 to 512 channels. Each block includes two 3 × 3 convolution layers (stride = 1, padding = 1) with LeakyReLU activations applied after each convolution. Following each convolutional block, spatial resolution is downsampled by a factor of two using a 2 × 2 max pooling layer (stride = 2). Conversely, the decoder path (red blocks) includes four up-convolution blocks that employ bilinear upsampling (scaling factor = 2) to progressively recover the spatial resolution back to 256 × 256. In particular, feature maps from corresponding encoder stages are concatenated to the decoder blocks through skip connections. This U-Net architecture facilitates the preservation of low-level spatial information through skip connections and high-level semantic representations through deeper layers, resulting in effective artifact suppression. The final output is produced by a 1 × 1 convolution followed by a tanh activation, which normalizes the reconstructed I/Q values to the range of [−1, 1]. The network was trained using the AdamW optimizer [28] with a learning rate schedule cycling between 1× 10^−4^ and 1 × 10^−7^ every 20,000 steps, and a batch size of 1 for 40,000 epochs. The batch size was fixed at 1 to preserve the acquisition-specific angular coherence modeled by the coherence loss; using larger batches would mix data from different acquisitions, including simulation, phantom, and in vivo datasets, and distort their distinct physical and scattering characteristics. The 40,000 epochs correspond to approximately 250 k training steps, during which the model exhibits rapid early convergence followed by a stable plateau, as shown in Figure 3. All experiments were conducted on an A6000 GPU (NVIDIA Corp., Santa Clara, CA, USA) with 48 GB of memory.

### 2.4. Experimental Setup

#### 2.4.1. Data Preparation

To train and evaluate the proposed network, we utilized two publicly available datasets, i.e., PICMUS (Plane-wave Imaging Challenge in Medical UltraSound) [29] and CUBDL (Challenge on Ultrasound Beamforming with Deep Learning) [30], to ensure diverse imaging conditions. As summarized in Table 1, the combined dataset consists of 79 sequences in total, including 2 simulation, 64 phantom, and 13 in vivo sequences. In addition, the dataset encompasses a range of acquisition parameters, such as different span angles (e.g., [−16, 16]° for PICMUS and [−8, 8]° for JHU) and different numbers of PW angles in a set of PWI data (e.g., 75-PWs for PICMUS and 31-PWs for TSH). For each sequence, all frames except for one validation frame and one test frame were used for training. The same trained model was then applied to the simulation, phantom, and in vivo test datasets.

For preprocessing, all radio-frequency (RF) data were pre-beamformed using a pixel grid–based phase-rotation delay calculator [31]. In this process, a pixel grid associated with the physical sizes (e.g., dx = dy = λ/2.5) is predefined, and delays in the complex domain are computed from in-phase/quadrature (I/Q) signals obtained via quadrature demodulation. As a result, two-channel I/Q images with consistent physical dimensions across different ultrasound acquisitions were generated, as shown in Figure 1a. The input and output data for the network were sized at 2 × 256 × 256 using random cropping with a patch size of 256 × 256, and Z-score normalization [32] was applied. A left–right flipping of the data was also performed to enhance data variability.

#### 2.4.2. Comparison Methods and Evaluation Metrics

To compare the performance across different beamforming methods, the conventional delay-and-sum (DAS) beamformer [33] was employed using single-PW (low-quality) and 75-PW (high-quality) transmissions. In addition, a representative rule-based adaptive beamformer, i.e., the delay-multiply-and-sum (DMAS) [10], was implemented and applied to single-PW data. For DL-based beamforming, a conventional unsupervised method using the deep coherence loss without weighting (DL-DCL) was compared against the proposed adaptive DCL approach employing either linear $\left(\alpha_{\mathrm{linear}}\right)$ or nonlinear $\left(\alpha_{\mathrm{nonlinear}}\right)$ weighting schemes, using single-PW data. For visualization of all B-mode images, the dynamic range was fixed at 60 dB to ensure a consistent and appropriate qualitative assessment, particularly with respect to soft-tissue contrast, speckle and artifact visibility, and the prevention of saturation at highly reflective boundaries [34,35].

To quantitatively evaluate the performance of the proposed method, the Full Width at Half Maximum (FWHM) and Peak Range Side-Lobe Level (PRSLL) were measured from the lateral intensity profiles. The FWHM was computed by measuring the width of the point spread function (PSF) at half of its peak amplitude, serving as an indicator of spatial resolution. The PRSLL quantifies the level of sidelobe artifacts and is defined as the ratio between the maximum sidelobe amplitude and the main-lobe peak amplitude, expressed in decibels (dB), as follows:

(3) $${PRSLL\;(\mathrm{dB})=\;20log}_{10}\left(\frac{A_{\mathrm{sidelobe},\max}}{A_{\mathrm{mainlobe},\max}}\right)$$

Therefore, a lower PRSLL value indicates greater suppression of sidelobe components. For evaluating contrast resolution, both Contrast-to-Noise Ratio (CNR) and generalized CNR (gCNR) were measured. The CNR [36,37] can be computed as:

(4) $${\mathrm{CNR}\;(\mathrm{dB})=20log}_{10}\frac{\left|\mu_{i}-\mu_{o}\right|}{\sqrt{\sigma_{i}^{2}+\sigma_{o}^{2}}}$$

where $\mu_{i}$ and $\mu_{o}$ denote the mean intensities of the background and anechoic regions, respectively, and $\sigma_{i}^{2}$ and $\sigma_{o}^{2}$ represent the corresponding intensity variances. The gCNR [36] quantifies the degree of overlap between the intensity distributions of the two regions, yielding a value between 0 and 1, where values closer to 1 indicate better separation between the background and anechoic regions:

(5) $$\mathrm{gCNR}=1-\int \min\{d_{i}\left(x\right),d_{o}(x)\}dx$$

where $d_{i}\left(x\right)$ and $d_{o}\left(x\right)$ represent the probability density functions of the background and anechoic region, respectively.

## 3. Results

### 3.1. Training & Validation Curve Analysis

To evaluate the training performance of the DL-based approaches (i.e., DL-DCL, DCL-A with $\alpha_{\mathrm{linear}}$, and DCL-A with $\alpha_{\mathrm{nonlinear}}$), we first examined the convergence behavior of their respective loss curves. Figure 3 shows the normalized training and validation loss curves as a function of training steps, with each step corresponding to a single gradient update, for the two proposed DCL-A methods (with $\alpha_{\mathrm{linear}}$ and $\alpha_{\mathrm{nonlinear}}$) compared with the conventional DL-DCL method. As shown in Figure 3a, all three DL-based methods exhibited stable convergence without abrupt fluctuations in their training loss trajectories, indicating that the network models were successfully trained. The validation loss consistently decreases along with the training loss, indicating that the models generalize well to unseen data, as depicted in Figure 3b. Notably, the proposed DCL-A models (both $\alpha_{\mathrm{linear}}$ (purple) and $\alpha_{\mathrm{nonlinear}}$ (green) schemes) show smoother convergence behavior and achieve lower final validation losses compared to the conventional DL-DCL (orange). This suggests that the adaptive weighting strategy effectively enhances training stability. Across all models, the gap between training and validation losses remains small throughout training, and no divergence is observed in later stages. This pattern indicates that neither overfitting nor underfitting occurs.

### 3.2. Simulation Study

**Figure 4:**
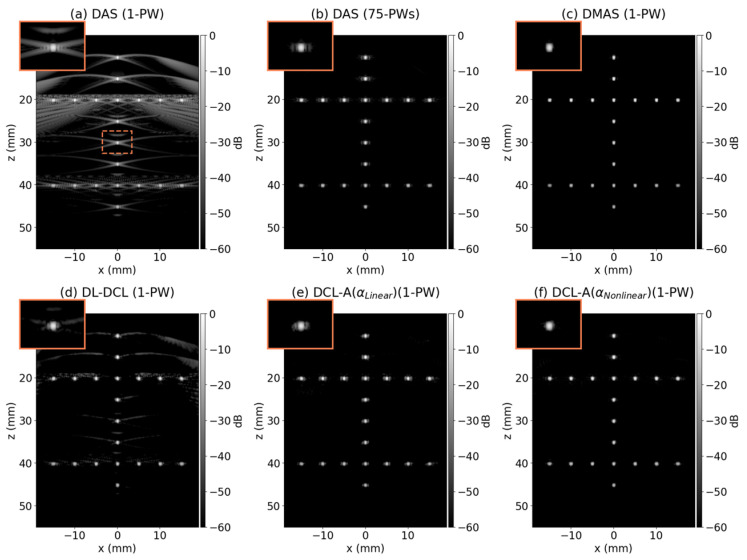
Reconstructed B-mode images (dynamic range = 60 dB) of the simulated point-target data from the PICMUS dataset using: (**a**) DAS with a single PW, (**b**) DAS with 75 PWs, (**c**) DMAS, (**d**) DL-DCL, (**e**) DCL-A with $\alpha_{\mathrm{linear}}$, and (**f**) DCL-A with $\alpha_{\mathrm{nonlinear}}$. The central target located at 30 mm was enlarged as shown in the dotted orange box in (**a**).

Figure 4a–f represents the reconstructed B-mode images (dynamic range = 60 dB) of the simulated point-target data from the PICMUS dataset using DAS with a single PW, DAS with 75 PWs, DMAS, DL-DCL, DCL-A with $\alpha_{\mathrm{linear}}$, and DCL-A with $\alpha_{\mathrm{nonlinear}}$, respectively. As shown in Figure 4a,b, traditional DAS with 75 PWs showed significantly improved image quality compared to DAS with a single PW, which suffers from severe grating lobe and sidelobe artifacts. The single-PW DMAS beamformer exhibited substantial performance in sidelobe suppression compared to DAS with 75 PWs, as illustrated in Figure 4c. Among the three DCL-based beamformers, as shown in Figure 4d–f, the DCL-A with $\alpha_{\mathrm{nonlinear}}$ achieved the best image quality, outperforming both the conventional DL-DCL and the DCL-A with $\alpha_{\mathrm{linear}}$. In contrast, the DL-DCL and the DCL-A with $\alpha_{\mathrm{linear}}$ still exhibited noticeable grating-lobe and sidelobe artifacts.

**Figure 5:**
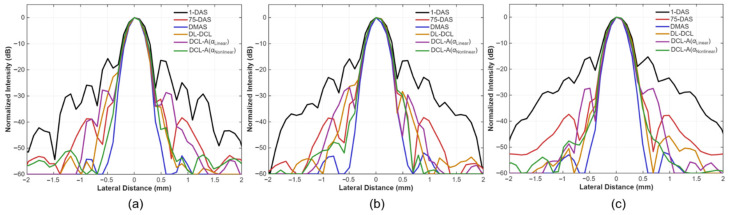
Normalized lateral profiles of the six beamforming methods for the three central point targets located at depths of (**a**) 20 mm, (**b**) 30 mm, and (**c**) 40 mm shown in Figure 4.

The lateral profiles of the six beamforming methods (DAS with a single PW, DAS with 75 PWs, DMAS, DL-DCL, DCL-A with $\alpha_{\mathrm{linear}}$, and DCL-A with $\alpha_{\mathrm{nonlinear}}$) were further analyzed. Figure 5a–c illustrates the normalized lateral profiles for the three central point targets at depths of 20 mm, 30 mm, and 40 mm, respectively. As shown in (Figure 5a–c), the rule-based DMAS beamformer achieved the best overall performance among the six methods, although DAS with 75 PWs also demonstrated strong sidelobe suppression. Among the three DCL-based methods, DCL-A with $\alpha_{\mathrm{nonlinear}}$ (green) showed the lowest sidelobe levels compared to conventional DL-DCL (orange) and DCL-A with $\alpha_{\mathrm{linear}}$ (purple).

Table 2 lists the measured FWHM and PRSLL values for the six comparison methods at the same point targets shown in Figure 5. For the FWHM measurements, the DMAS beamformer achieved the best lateral resolution for all point targets, exhibiting the narrowest mainlobe width (0.32 mm in average). The three DCL-based methods (DL-DCL, DCL with $\alpha_{\mathrm{linear}}$, and DCL with $\alpha_{\mathrm{nonlinear}}$) and DAS with 75 PWs resulted in comparable lateral resolutions with similar FWHM values (about 0.38 mm). In contrast, DAS with a single PW produced the poorest lateral resolution across all depths. For the PRSLL measurements, DMAS and the DCL-A with $\alpha_{\mathrm{nonlinear}}$ demonstrated the lowest sidelobe levels among the six beamforming methods (under −42 dB). Notably, the proposed DCL-A with $\alpha_{\mathrm{nonlinear}}$ achieved a substantial reduction in sidelobe artifacts compared to the conventional DL-DCL method, i.e., approximately 6 dB difference, attributable to the adaptive weighting strategy.

### 3.3. Phantom Study

**Figure 6:**
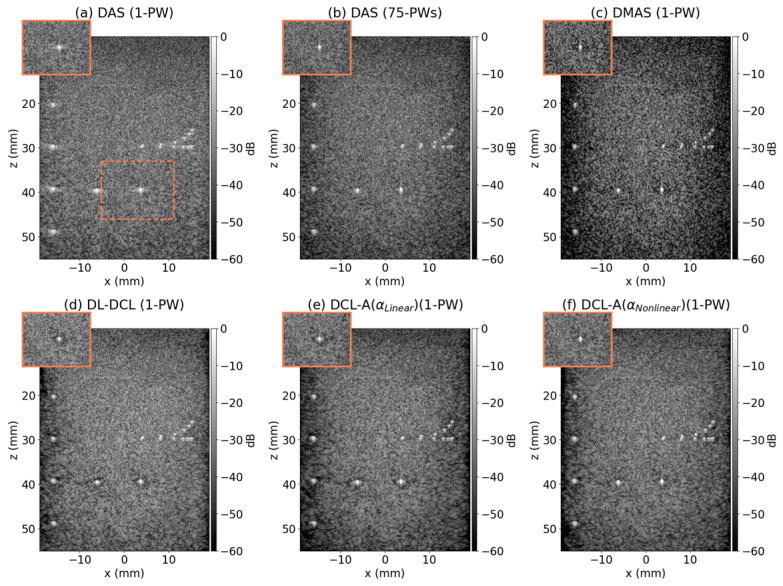
Reconstructed B-mode results of the point target phantom from CUBDL dataset using: (**a**) DAS with a single PW, (**b**) DAS with 75 PWs, (**c**) DMAS, (**d**) DL-DCL, (**e**) DCL-A with $\alpha_{\mathrm{linear}}$, and (**f**) DCL-A with $\alpha_{\mathrm{nonlinear}}$. The target indicated by the dotted orange box in (**a**) was enlarged.

Figure 6 represents the reconstructed B-mode results (dynamic range = 60 dB) from CUBDL datasets using the six beamforming techniques (i.e., DAS with a single PW, DAS with 75 PWs, DMAS, DL-DCL, DCL-A with $\alpha_{\mathrm{linear}}$, and DCL-A with $\alpha_{\mathrm{nonlinear}}$). As shown in Figure 6, the DAS beamformer using a single PW exhibited low image quality due to high sidelobe levels, although the associated grating-lobe and sidelobe artifacts were largely obscured by background speckles. The sidelobe artifacts presented in the single-PW DAS result were markedly suppressed by employing multiple PW transmissions, with DAS using 75 PWs yielding a substantial improvement in overall image quality, as illustrated in Figure 6b. Figure 6c shows the result of DMAS beamforming, which effectively suppressed sidelobe artifacts. However, it introduces dark region artifacts (DRAs) [11] that reduce the dynamic range, potentially limiting its suitability for clinical use due to the loss of clinically relevant information. Among the DCL-based deep beamforming techniques, DCL-A with $\alpha_{\mathrm{nonlinear}}$ outperformed both the conventional DL-DCL and DCL-A with $\alpha_{\mathrm{linear}}$ in suppressing sidelobe artifacts, while maintaining comparable mainlobe widths.

Table 3 lists the measured lateral FWHM and PRSLL values using the six beamforming methods for the three point targets located at 40 mm shown in Figure 6. For the FWHM measurement, both DAS with 75 PWs and the DMAS beamformer exhibited the highest lateral resolution, yielding the lowest FWHM value of approximately 0.60 mm. In contrast, DAS with a single PW showed the lowest lateral resolution, with the highest FWHM value of approximately 0.96 mm. The three DCL-based beamforming methods exhibited comparable FWHM values, all of which provided higher lateral resolution than DAS with a single PW. In the PRSLL measurement, DCL-A with $\alpha_{\mathrm{nonlinear}}$ exhibited the lowest value (i.e., −26.02 dB) among all six methods except for DMAS, which suffers from DRAs. DCL-A with $\alpha_{\mathrm{linear}}$ exhibited a relatively higher PRSLL compared to the other DCL-based beamformers (DL-DCL and DCL-A with $\alpha_{\mathrm{nonlinear}}$), while achieving the best FWHM performance among the three.

### 3.4. In Vivo Study

**Figure 7:**
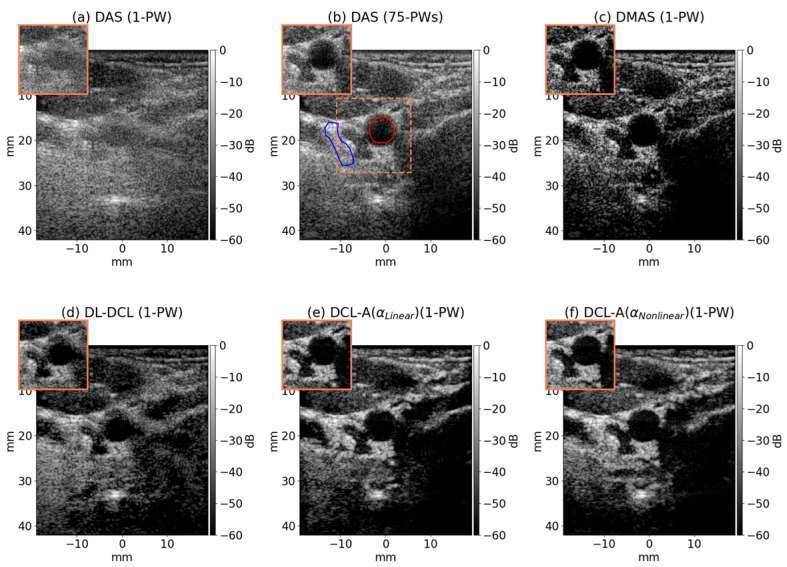
In vivo carotid artery B-mode images reconstructed using (**a**) DAS with a single PW, (**b**) DAS with 75 PWs, (**c**) DMAS, (**d**) DL-DCL, (**e**) DCL-A with $\alpha_{\mathrm{linear}}$, and (**f**) DCL-A with $\alpha_{\mathrm{nonlinear}}$ from the PICMUS dataset. The marked areas (red and blue) in (**b**) indicate anechoic and background regions used to measure contrast resolution (CNR and gCNR). The target indicated by the dotted orange box in (**b**) was enlarged.

The six beamforming methods (DAS with a single PW, DAS with 75 PWs, DMAS, DL-DCL, DCL-A with $\alpha_{\mathrm{linear}}$, and DCL-A with $\alpha_{\mathrm{nonlinear}}$) were evaluated on clinical in vivo data of the carotid artery in cross-sectional view from the PICMUS dataset. Figure 7a–f illustrates the B-mode results using the six comparisons. As shown in Figure 7a,b, DAS with 75 PWs exhibited substantially improved image quality compared to DAS with a single PW, where anatomical features such as the carotid vessel and thyroid nodule were obscured by severe artifacts. DMAS beamforming delineated clear margin sharpness and tissue boundaries by greatly suppressing beamforming artifacts, as depicted in Figure 7c. However, severe DRAs occurred, making it difficult to distinguish between the anechoic (vascular) region and the hypoechoic (muscle or surrounding tissue) region. Among the three DCL-based deep learning methods, as illustrated in Figure 7d–f, DCL-A with $\alpha_{\mathrm{nonlinear}}$ exhibited the highest image quality compared to DL-DCL and DCL-A with $\alpha_{\mathrm{linear}}$, with clear margin sharpness and improved contrast due to reduced beamforming artifacts.

Table 4 lists the measured CNR and gCNR values for the cross-sectional carotid vessel region and its surrounding tissue from the in vivo data, as also illustrated in Figure 7b. As listed in Table 4, the proposed DCL-A with $\alpha_{\mathrm{nonlinear}}$ exhibited the highest CNR and gCNR values among the 6 comparisons, i.e., 5.70 dB and 0.97, while the three unsupervised beamforming techniques (DL-DCL, DCL-A with $\alpha_{\mathrm{linear}}$, and DCL-A with $\alpha_{\mathrm{nonlinear}}$) produced comparable CNR and gCNR values. For the three rule-based traditional beamforming methods (DAS with a single PW, DAS with 75 PWs, an DMAS), the DAS with 75 PWs showed the higher CNR and gCNR values than DAS with a single PW and DMAS. Interestingly, DMAS beamforming exhibited much lower contrast resolution, as DRAs increased speckle variance and pixel discontinuity.

## 4. Discussion

In this study, we identified a key limitation of the conventional unsupervised DL-DCL beamformer: its reliance on a uniform weighting strategy in the coherence loss function, which treats all plane-wave angle pairs equally. We hypothesized that this approach is suboptimal, as it does not distinguish between main-lobe-correlated large-angle pairs and sidelobe-correlated small-angle pairs. To address this limitation, we proposed the DCL-A framework, which incorporates an adaptive weighting function, α(d), based on the frame (angular) distance.

Our experimental results demonstrates the hypothesis. The most notable finding arose from the comparison between our two proposed strategies. While the DCL-A ($\alpha_{\mathrm{linear}}$) method achieved competitive FWHM values, its performance in sidelobe suppression (PRSLL) was limited, showing values significantly worse than the baseline DL-DCL (Table 2 and Table 3). This indicates that simply applying a weight is insufficient; the design of the weighting function is critical (e.g., nonlinear weight).

In contrast, the DCL-A ($\alpha_{\mathrm{nonlinear}}$) strategy proved highly effective. This method consistently maintained competitive FWHM values (comparable to the DAS with 75-PWs) while achieving markedly superior sidelobe suppression (PRSLL). As shown in Table 2 and Table 3, its PRSLL values were significantly better than other methods, including the DAS with 75-PWs. These results suggest that the nonlinear weighting function (Figure 1c), which strongly penalizes small-angle pairs while favoring large-angle pairs, provides an optimal balance for suppressing sidelobe artifacts without compromising main-lobe resolution.

Furthermore, the clinical relevance of our findings was demonstrated in both phantom and in vivo studies. While DMAS often shows numerically competitive metrics (e.g., FWHM in Table 2, PRSLL in Table 3), these values can be misleading. As visually confirmed (Figure 6c and Figure 7c) and reflected in its poor CNR (Table 4), DMAS introduces severe dark region artifacts (DRAs) that disrupt natural speckle texture and reduce diagnostic value. Our DCL-A ($\alpha_{\mathrm{nonlinear}}$) method overcomes this limitation. Notably, it preserves speckle texture (Figure 6f) without DRAs and achieves higher CNR/gCNR values than the DAS with 75 PWs (Table 4). This result highlights the clear potential of our adaptive framework, achieving high contrast resolution and high spatiotemporal resolution while avoiding DRAs.

Our proposed DCL-A ($\alpha_{\mathrm{nonlinear}}$) function employed a specific exponential base (Equation (1)), which was chosen empirically. A more exhaustive search for the optimal weighting function, such as other exponential bases, quadratic functions, or even a learnable function, is under investigation. Given the relatively low performance of the DCL-A ($\alpha_{\mathrm{linear}}$) model, it is evident that the shape of the weighting function is a critical hyperparameter, and further optimization could lead to even better results. Future work will address these limitations, including expanding our dataset to explore more advanced network architectures and conducting a systematic study to optimize the adaptive weighting function itself. For example, an attention-based learnable adaptive weighting scheme or a data-driven hyperparameter optimization strategy could further enhance performance while simultaneously reducing reliance on empirical parameter selection.

The studies presented in this work should be further extended to a broader spectrum of conditions, including variations in imaging depth, frequency bands, and clinical environments, to more comprehensively assess the generalizability and robustness of the proposed method. To this end, future evaluations will incorporate different imaging probes (e.g., convex and phased arrays) applied to various anatomical regions, as these probes operate at greater imaging depths and lower center frequencies compared to linear array transducers.

In terms of computational time for real time beamforming, DAS with 1 PW shows the lowest computational time, achieving 11 frames-per-seconds (FPS), whereas DAS with 75 PWs and DMAS with 1 PW were considerably slower, at approximately 0.2 FPS, due to the large number of PW frames and high computational complexity [38]. The three unsupervised beamforming approaches (conventional DCL, DCL-A with $\alpha_{\mathrm{linear}}$, and DCL-A with $\alpha_{\mathrm{nonlinear}}$) demonstrated similar inference speeds, yielding substantially faster processing than DAS with 75-PWs or DMAS with 1-PW, at approximately 3.7 FPS (inference time ~270 ms). Despite this improvement, these DL-based methods remain limited for real time imaging; employing a lightweight neural network for hardware acceleration [39] may further improve inference speed.

## 5. Conclusions

In conclusion, the proposed DCL-A framework, particularly with the nonlinear weighting function, effectively addresses the limitations of conventional SPWI by adaptively emphasizing large-angle, main-lobe-correlated PW pairs and suppressing small-angle, sidelobe-correlated pairs. This approach not only significantly reduces beamforming artifacts such as sidelobes and grating lobes but also maintains competitive lateral resolution (FWHM) and achieves superior contrast resolution (CNR and gCNR) across simulation, phantom, and in vivo datasets. By improving image quality without compromising the ultrafast acquisition rate, the DCL-A method demonstrates substantial potential for enhancing diagnostic accuracy in clinical ultrasound applications.

## Figures and Tables

**Table 1:** Summary of publicly available datasets used for training and validation.

Data Source		Simulation	Phantom	In Vivo	PW Span Angle (°)	Number of PW Angles	Number of Sequences	Number of Frames	
								Training	Validation/Test
PICMUS [29]		2	2	2	[−16, 16]	75	6	438	6
CUBDL [30]	INS	-	5	-	[−16, 16]	75	5	365	5
	MYO	-	5	-	[−15, 15]	75	5	365	5
	UFL	-	2	-	[−15, 15]	75	2	146	2
	JHU	-	-	11	[−8, 8]	75 (73)	11	803	11
	TSH	-	50	-	[−15, 15]	31	50	1450	50
Total		2	64	13	-	-	79	3567	79

**Table 2:** Measured lateral FWHM and PRSLL values for the six beamforming methods at the central point targets located at depths of 20, 30, and 40 mm.

Metrics	Depth	DAS (1-PW)	DAS (75-PWs)	DMAS	DL-DCL	DCL-A $(\alpha_{Linear}$)	DCL-A $(\alpha_{Nonlinear}$)
FWHM [mm]	20 mm	0.42	0.37	0.31	0.34	0.36	0.37
	30 mm	0.44	0.38	0.32	0.38	0.36	0.37
	40 mm	0.46	0.40	0.33	0.42	0.36	0.40
	Mean ± STD	0.44 ± 0.02	0.38 ± 0.02	0.32 ± 0.01	0.38 ± 0.04	0.36 ± 0.00	0.38 ± 0.02
PRSLL [dB]	20 mm	−16.36	−31.25	−39.81	−34.23	−28.67	−42.18
	30 mm	−16.67	−30.38	−46.25	−28.37	−29.59	−39.47
	40 mm	−15.24	−36.30	−48.65	−45.66	−27.41	−46.94
	Mean ± STD	−16.09 ± 0.75	−32.64 ± 3.20	−44.90 ± 4.57	−36.09 ± 8.79	−28.56 ± 1.09	−42.86 ± 3.78

**Table 3:** Measured lateral FWHM and PRSLL values (Mean ± STD) using the six beamforming methods for the three point targets located at 40 mm shown in Figure 6.

	DAS (1-PW)	DAS (75-PWs)	DMAS	DL-DCL	DCL-A $(\alpha_{Linear}$)	DCL-A $(\alpha_{Nonlinear}$)
FWHM [mm]	0.96 ± 0.45	0.61 ± 0.18	0.57 ± 0.13	0.65 ± 0.15	0.62 ± 0.22	0.64 ± 0.11
PRSLL [dB]	−11.86 ± 3.00	−18.86 ± 5.78	−27.38 ± 12.82	−18.55 ± 4.26	−15.07 ± 3.03	−26.02 ± 4.59

**Table 4:** CNR and gCNR measurements for the in vivo study.

	DAS (1-PW)	DAS (75-PWs)	DMAS	DCL	DCL-A $(\alpha_{Linear}$)	DCL-A $(\alpha_{Nonlinear}$)
CNR [dB]	1.61	4.43	3.15	5.68	5.65	5.70
gCNR	0.62	0.96	0.89	0.95	0.94	0.97

## Data Availability

The datasets used in this study are publicly available. The PICMUS dataset is available at https://www.creatis.insa-lyon.fr/Challenge/IEEE_IUS_2016/ (accessed on 2 September 2024), and the CUBDL dataset is available at https://cubdl.jhu.edu/ (accessed on 12 September 2024).
